# The Subcellular Compartmentalization of Arginine Metabolizing Enzymes and Their Role in Endothelial Dysfunction

**DOI:** 10.3389/fimmu.2013.00184

**Published:** 2013-07-09

**Authors:** Feng Chen, Rudolf Lucas, David Fulton

**Affiliations:** ^1^Vascular Biology Center, Georgia Regents University, Augusta, GA, USA; ^2^Department of Pharmacology, Georgia Regents University, Augusta, GA, USA

**Keywords:** eNOS, l-arginine, nitric, arginase, CAT-1, ASL, ASS, l-citrulline

## Abstract

The endothelial production of nitric oxide (NO) mediates endothelium-dependent vasorelaxation and restrains vascular inflammation, smooth muscle cell proliferation, and platelet aggregation. Impaired production of NO is a hallmark of endothelial dysfunction and promotes the development of cardiovascular disease. In endothelial cells, NO is generated by endothelial nitric oxide synthase (eNOS) through the conversion of its substrate, l-arginine to l-citrulline. Reduced access to l-arginine has been proposed as a major mechanism underlying reduced eNOS activity and NO production in cardiovascular disease. The arginases (Arg1 and Arg2) metabolize l-arginine to generate l-ornithine and urea and increased expression of arginase has been proposed as a mechanism of reduced eNOS activity secondary to the depletion of l-arginine. Indeed, supplemental l-arginine and suppression of arginase activity has been shown to improve endothelium-dependent relaxation and ameliorate cardiovascular disease. However, this simple relationship is complicated by observations that l-arginine concentrations in endothelial cells remain sufficiently high to support NO synthesis. Accordingly, the subcellular compartmentalization of intracellular l-arginine into poorly interchangeable pools has been proposed to allow for the local depletion of pools or pockets of l-arginine. In agreement with this, there is considerable evidence supporting the importance of the subcellular localization of l-arginine metabolizing enzymes. In endothelial cells *in vitro* and *in vivo*, eNOS is found in discrete intracellular locations and the capacity to generate NO is heavily influenced by its localization inside the cell. Arg1 and Arg2 also reside in different subcellular environments and are thought to differentially influence endothelial function. The plasma membrane solute transporter, CAT-1 and the arginine recycling enzyme, arginosuccinate lyase, co-localize with eNOS and facilitate NO release. Herein, we highlight the importance of the subcellular location of eNOS and arginine transporting and metabolizing enzymes to NO release and cardiovascular disease.

## Endothelial Dysfunction

The past three decades have provided unprecedented gains in our understanding of vascular biology. It is now hard to conceive of a time when the vascular endothelium was thought to be a simple barrier, an inert layer of cells lining the lumen of blood vessels. However this was the prevailing view prior to 1981 and the world of vascular biology was irrevocably changed with Furchgott’s discovery of an ability of the endothelium to direct changes in vasomotor function (1). In the time since, the depth and pace of research to understand the myriad functions of the endothelium has been remarkable. Not the least of these has been the discovery of endothelial nitric oxide synthase (eNOS) (2–4), an enzyme selectively expressed in the endothelial cells with the ability to generate nitric oxide (NO) and thus regulate blood vessel tone (5). Dysfunction of the vascular endothelium is considered to be the harbinger of cardiovascular disease and precedes the development of overt symptoms (6, 7). Given the importance of eNOS and endogenous NO production to endothelial function, it is not surprising that considerable effort has been focused on the mechanisms influencing eNOS activity in cardiovascular disease. The primary enzymatic function of eNOS is to catalyze the NADPH-dependent conversion of l-arginine into NO, a process shared by the two other NOS isoforms (8). Once formed, NO has an expansive array of cellular targets both locally in the endothelium to influence inflammatory signaling, metabolism, exocytosis, proliferation, motility, and survival, but also in adjacent cells such as vascular smooth muscle cells to decrease vasomotor tone, proliferation and migration, and in platelets to suppress aggregation (9). Loss of these functions promotes increased inflammation, thrombosis, high blood pressure, and vascular cell proliferation, processes that are intimately involved in the development of cardiovascular disease.

## l-Arginine

Because of the obligatory role of l-arginine in NO synthesis, considerable attention has been focused on the importance of l-arginine availability in the vascular production of NO. Fueling this interest were early studies reporting that l-arginine could directly stimulate EDRF/NO synthesis (10–12) and that compromised endothelial function in cardiovascular disease states could be improved by supplementation with l-arginine both in animals (13–17), healthy humans (18) and those with high cholesterol (19–21), cardiac transplantation (22), peripheral artery disease (23), pulmonary hypertension (24), and angina (25). Considerable evidence pointed toward l-arginine deficiency being a major rate limiting step in the synthesis of NO. However, the affinity of eNOS for l-arginine is low (∼2–3 μM) (26) and the amount of l-arginine in endothelial cells is hundreds of times higher (∼840 μM) (27) suggesting that a substrate deficiency was an unlikely unitary cause of eNOS dysfunction and that additional mechanisms of dysfunction must exist.

## Subcellular Localization of eNOS

The co-translational *N*-myristoylation (glycine 2) and post-translational cysteine palmitoylation of eNOS (cysteines 15 and 26) enable membrane binding and the discrete subcellular targeting (28). In the endothelial cell, eNOS can be found predominantly localized to the perinuclear Golgi (29) and microdomains of the plasma membrane, including caveolae and lipid rafts (30, 31). eNOS has also been reported in other compartments, such as the mitochondria, the nucleus and the cytoskeleton (32, 33). The importance of location to eNOS function and cellular NO release was first demonstrated by mutations that prevent both myristoylation and palmitoylation resulting in an enzyme that is catalytically competent in activity assays outside the cell, but exhibits dramatically reduced capacity to generate NO in intact cells (29). Furthermore, the relative activity of eNOS varies depending on its intracellular location with the highest activity observed from eNOS at the plasma membrane, followed by outer membranes of the cis-Golgi and very low activity in the cytosol, nucleus, and mitochondria (32, 34, 35). Given the dramatic ability of subcellular location to influence eNOS activity and NO release, it is not surprising that compartmentalization has been proposed as a major mechanism by which the local concentration of l-arginine can influence NO release (36).

## l-Arginine Transporters

The concentration of l-arginine in human plasma is ∼100–200 μM (37) and higher concentrations, up to 840 μM (27) can be found within the endothelial cell reflecting the existence of transport processes. A number of distinct transmembrane transporters exist on the plasma membrane of endothelial cells that mediate the predominantly sodium independent import of l-arginine via y+ and y+l transporters. The major genes involved in y+ import are CAT-1 (SLC7A1) and CAT-2 (SLC7A2), whereas for y+L import, LAT1 (SLC7A7 and SLC3A2) and LAT2 (SLC7A6 and SLC3A2) are required (36). The presence of CAT-1 in plasmalemma caveolae and the ability of extracellular l-arginine to stimulate NO release in cells with abundant l-arginine levels has led to the hypothesis that l-arginine exists in poorly interchangeable subcellular compartments and reaches eNOS in sufficient concentrations via metabolite channeling (36). While there is suggestive data for the existence of these pools (38), direct evidence and a mechanism for l-arginine sequestration is lacking. A further wrinkle to this story is that the cationic amino acid transporter, CAT-1 can stimulate eNOS activity via direct binding rather than delivering abundant l-arginine to its catalytic doorstep (39).

## l-Arginine Recycling

Endothelial cells can maintain their l-arginine levels despite the continuous release of NO (40), suggesting the existence of mechanisms to recover substrate. Indeed, l-citrulline, the byproduct of eNOS-dependent NO generation, can be converted back to l-arginine via the sequential actions of arginosuccinate synthase (ASS) and arginosuccinate lyase (ASL) (41). The co-localization of ASS/ASL with eNOS in plasma membrane caveolae suggests that l-arginine recycling from l-citrulline is a significant source of NO (42, 43). The importance of this pathway is revealed by impaired endothelium-dependent NO generation and increased blood pressure in humans and mice with ASL deficiency (44). Not only do ASL and ASS co-localize with eNOS, but they have been shown to bind directly and regulate eNOS activity (44). A caveat to these studies is that only a fraction of eNOS is present in plasma membrane caveolae and significant amounts of eNOS can be found on endomembranes such as the Golgi. It is not yet known if eNOS at the Golgi or other organelles are regulated by arginine regulatory enzymes in the same way as the plasma membrane/caveolae bound eNOS. l-arginine can also be generated from the breakdown of proteins via both proteosomal and lysosomal pathways, which liberates l-arginine (45). The breakdown of proteins also liberates asymmetric methylated arginines, monomethylarginine (MMA), and asymmetric dimethylarginine (ADMA) which are potent substrate inhibitors of eNOS activity. The methylation of proteins is increased in cardiovascular disease, providing a source for the increased levels of MMA and ADMA via proteolysis (46). Methylated arginines are metabolized by the dimethylarginine dimethylaminohydrolases (DDAH1 and DDAH2). DDAH is found primarily in the cytosol (47) although there are reports of expression in the mitochondria (47) and nucleus (48). Accumulation of asymmetric methylated arginines results in a degree of eNOS-inhibition that is proportional to the ratio of l-arginine/methylated arginine. The inhibition of eNOS can be relieved by supplementation with l-arginine leading to increased production of NO and improvement of endothelial function (49).

## Arginases

Arginase I and Arginase II are homologous genes encoded by different chromosomes that share the catalytic function of converting l-arginine into urea and ornithine (50). A significant difference between Arginase I and Arginase II is their distinct subcellular distribution, with Arginase I detected predominantly in the cytosol and Arginase II within the mitochondria (51, 52). As enzymes that consume the substrate for eNOS, l-arginine, the arginases have been proposed as endogenous antagonists of eNOS. Increased expression and activity of Arginase I have been implicated in numerous cardiovascular diseases including diabetic retinopathy, asthma, coronary artery dysfunction during renovascular hypertension, and sickle cell disease (53–57) and Arginase II has been shown to be specifically increased in retinopathy of prematurity, human pulmonary arterial endothelial cells during hypertension, atherosclerosis, and in diabetic renal injury (57–60). Numerous studies have shown that increased expression of arginase correlates with impaired NO synthesis and that inhibition of arginase increases NO production (53, 61, 62). However, this seemingly simple relationship between eNOS and the arginases is complicated by enzyme kinetics and l-arginine concentrations. The affinity of eNOS for arginine is relatively high (*K*_m_ = 3 μM), the affinity of arginase for l-arginine relatively low (2 mM) and the concentrations of intracellular l-arginine (300–800 μM) sufficient to support near maximal eNOS activity. Two explanations have been proposed to explain the inhibitory actions of arginase, one is the 1000-fold higher enzyme activity (*V*_max_) and the other, the compartmentalization and regional deficiency of l-arginine (63). Vascular dysfunction achieved through the arginase-mediated depletion of l-arginine can be reversed with l-arginine supplementation (64) but this also drives increased arginase activity.

## l-Arginine Supplementation

The preceding evidence has emphasized the important role l-arginine plays in the maintenance of endothelial and cardiovascular function and is supported by studies showing that at least in the short-term; l-arginine supplementation can increase endothelial function and mitigate disease. However, more recent evidence suggests that chronic long term supplementation offers little benefit and may instead be harmful (65). The reasons for this are not well understood and likely to be numerous. Chronic exposure to high levels of NO can desensitize NO signaling, impair l-arginine import and increase vascular lesions and mortality (66–69). In contrast, inhibition of endogenous NO can increase sensitivity to NO donors and collectively this suggests that there is pushback when “pushing” NO signaling. Chronic supplementation with l-arginine can also influence other pathways including the greater activation of iNOS (70) which unlike eNOS, is primarily constrained by substrate availability, and the increased expression and catalytic activity of the arginases due to their higher *K*_m_. A consequence of increased arginase activity is the production of ornithine and attendant elevation of l-proline and the polyamines which can promote cell proliferation and maladaptive vascular remodeling (71).

## Conclusion

l-Arginine is a semi-essential amino acid with a number of important roles in the endothelium including the ability to drive NO production. The compartmentalization of arginine metabolizing and transporting enzymes has important ramifications for endothelial function and cardiovascular health. l-arginine transporters and recycling enzymes have been found in the same intracellular location as eNOS, and some have been found to directly bind eNOS. However, whether this proximity is necessary for providing eNOS with ready access to l-arginine is questionable. Catalytically inactive forms of ASL and substrate-inhibition of CAT-1 do not prevent the ability of these enzymes/transporters to stimulate NO release and this suggests they instead play a structural role in the activation of eNOS. The arginases, which compete for and metabolize l-arginine, particularly when l-arginine is in high abundance, do not reside in the same intracellular locations as eNOS (and presumably do not physically associate) and thus are unlikely to exclusively regulate l-arginine content in pools accessible to eNOS. Instead a more important role of the arginases may be to generate l-proline and polyamines that can negatively impact endothelial and vascular function. The accumulation of asymmetric methylated arginines occurs at the major sites of protein degradation, the proteasome and lysosome, and like the cytosolic DDAH, they are not thought to be in close proximity to eNOS. Frequently underappreciated is the important role eNOS subcellular location has on NO release. Targeting eNOS to the plasma membrane supports the highest levels of NO production followed by the Golgi and the cytoplasm (35). Rendering eNOS insensitive to calcium overrides the effects of intracellular location on eNOS activity and suggests that local calcium and not l-arginine, is the major determinant of efficient NO release (32, 72). While compartmentalization may not be a critical mechanisms by which l-arginine influences eNOS activity, its ability to increase NO release is well documented. However, the failure of supplemental l-arginine to improve cardiovascular health may be considered another lesson learned of why too much of a good thing can be bad. Chronic high levels of NO can result in the refractoriness of its targets to respond and is well documented in vascular smooth muscle. Mechanisms that temporarily restrict eNOS activity such as caveolin-1 or the location of eNOS on membranes of the Golgi enable efficient production of NO in the right amount at the right time for the right response.

## Conflict of Interest Statement

The authors declare that the research was conducted in the absence of any commercial or financial relationships that could be construed as a potential conflict of interest.
